# Peroxisome Proliferator-Activated Receptors and the Heart: Lessons from the Past and Future Directions

**DOI:** 10.1155/2015/271983

**Published:** 2015-10-26

**Authors:** Wang-Soo Lee, Jaetaek Kim

**Affiliations:** ^1^Division of Cardiology, Department of Internal Medicine, College of Medicine, Chung-Ang University, Seoul 06973, Republic of Korea; ^2^Division of Endocrinology and Metabolism, Department of Internal Medicine, College of Medicine, Chung-Ang University, Seoul 06973, Republic of Korea

## Abstract

Peroxisome proliferator-activated receptors (PPARs) belong to the nuclear family of ligand activated transcriptional factors and comprise three different isoforms, PPAR-*α*, PPAR-*β*/*δ*, and PPAR-*γ*. The main role of PPARs is to regulate the expression of genes involved in lipid and glucose metabolism. Several studies have demonstrated that PPAR agonists improve dyslipidemia and glucose control in animals, supporting their potential as a promising therapeutic option to treat diabetes and dyslipidemia. However, substantial differences exist in the therapeutic or adverse effects of specific drug candidates, and clinical studies have yielded inconsistent data on their cardioprotective effects. This review summarizes the current knowledge regarding the molecular function of PPARs and the mechanisms of the PPAR regulation by posttranslational modification in the heart. We also describe the results and lessons learned from important clinical trials on PPAR agonists and discuss the potential future directions for this class of drugs.

## 1. Introduction

Peroxisome proliferator-activated receptors (PPARs) belong to the nuclear receptor superfamily of ligand-activated transcription factors and include three member isoforms—*α*, *β*/*δ*, and *γ*—encoded by distinct genes located on different chromosomes with a high degree of interspecies sequence conservation [1–5]. Interestingly, while significant homology exists between PPAR proteins, they play distinct, functional roles in energy metabolism [5].

PPARs are subjected to transactivation or transrepression through distinct mechanisms, which lead to the induction or repression of target gene expression [1]. For this, PPARs dimerize with the retinoid receptor and subsequently bind sequence-specific promoter elements in their target genes to control several facets of normal cellular physiology as well as pathology. Disruption of this pathway contributes to disease progression in obesity, diabetes, and cancers. This occurs through regulation of growth and migration, apoptosis, fatty acid (FA) metabolism pathways, and oxidative stress responses. Moreover, PPARs are also known to regulate inflammatory processes that are linked to metabolic homeostasis in tissues, such as liver, adipose tissue, intestine, skeletal muscle, and cardiovascular system [1–9]. Importantly, each PPAR family member has distinct metabolic functions determined by their ligand affinity, expression, and activity, which are both tissue- and pathway-dependent [6].

All three PPAR isoforms are expressed in the heart; however, their roles in cardiac function and the outcomes of respective agonists in preclinical animal models and clinical trials vary immensely. Furthermore, studies of PPARs on myocardial fatty acid metabolism and cardiac function are currently being conducted. Thus, it is necessary to understand current PPAR research, as well as PPAR biology in the heart. In this review, we focus on the functions of PPARs in myocardial biology in addition to their regulatory effects on glucose and lipid metabolism, and we describe their potential clinical implications and future directions.

## 2. Molecular Structure of PPARs

**Figure 1:**
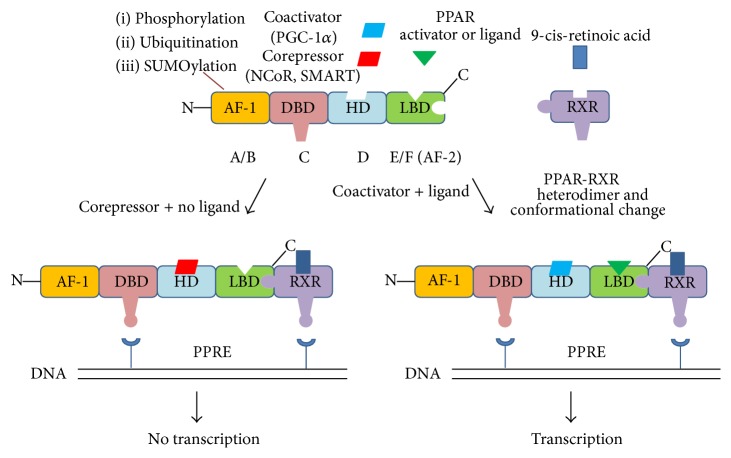
Structure of PPAR and its transactivation or transrepression process. In the absence of ligand, the PPAR-RXR heterodimer recruits corepressors (left process). When ligand binds, conformational changes in PPAR-RXR induce dissociation of corepressor complex. Active transcriptional complex assembles with coactivator proteins. PPAR binds to PPRE and assembles coactivator complexes (right process). PGC-1*α*: PPAR-*γ* coactivator 1*α*, NCoR: nuclear receptor corepressor, SMART: silencing mediator of retinoid and thyroid hormone receptor, AF: activation function, DBD: DNA-binding domain, HD: hinge domain, LBD: ligand-binding domain, RXR: retinoid X receptor, and PPRE: peroxisome proliferator response element.

PPARs are orphan nuclear receptors that belong to the thyroid, steroid, and retinoid hormone receptor superfamilies of ligand-activated nuclear hormone receptors [4, 5, 10–12]. After binding with their respective ligands, PPARs translocate to the nucleus, where they undergo a conformational change, interact with transcriptional cofactors, and regulate gene transcription [13–15]. PPAR isoforms possess five or six structural regions within four functional domains, termed A/B, C, D, and E/F (Figure 1) [6, 12]. The variable N-terminal, ligand-independent transactivation domain (A/B domain) contains an activation function- (AF-) 1 motif, which is a target of kinase phosphorylation [6, 12]. The 70-amino-acid PPAR DNA-binding domain (C domain) contains two highly conserved zinc finger motifs that facilitate binding to the peroxisome proliferator response element (PPRE) [6, 12]. The hinge region (D domain) acts as a docking site for cofactors. The C-terminal or ligand-binding domain (the E/F domain) is responsible for ligand specificity and the activation of PPAR binding to the PPRE, which increases target gene expression. The E/F domain uses cofactors for the transactivation via the ligand-dependent trans-AF-2 [6, 12]. When activated by endogenous or synthetic ligands, PPARs heterodimerize with the 9-cis-retinoic acid receptor (retinoid X receptor; RXR), triggering a conformational change and their nuclear translocation [6, 12]. The PPAR-RXR heterodimer then binds the PPRE in the target gene promoter region, subsequently altering coactivator/corepressor dynamics to modulate the transcription machinery controlling gene expression [6, 16–20]. In the past 20 years, many PPAR cofactors have been identified; however, the complete physiological functions of these molecules in receptor-, gene-, and/or cell-specific transcription remain to be elucidated [21].

## 3. Extracardiac Function of PPARs and Their Ligands

The first PPAR isoform to be cloned, PPAR-*α*, was identified in 1990 and its name of PPAR originated from its activation by peroxisome proliferator chemicals [22, 23]. The PPAR-*α* gene is located on human chromosome 22q12.2-13.1 [24], and its expression is highest in tissues with elevated FA oxidation rates—such as liver, heart, and skeletal muscle—where it functions as a major regulator of FA homeostasis [23–27]. PPAR-*α* is also highly expressed in brown adipose tissue, kidney, adrenal gland, and the majority of cell types, including macrophages, smooth muscle cells, and endothelial cells [6, 26–28]. Unsaturated/saturated FAs, leukotriene (LT) derivatives, and very low-density lipoprotein (VLDL) hydrolysis products are endogenous ligands that bind PPAR-*α* with the greatest affinity. Moreover, PPAR-*α* is a major regulator of the mitochondrial and peroxisomal *β*-oxidation pathways, which are reported to be involved in the pathogenesis of various liver complications—such as hepatocarcinogenesis in rodent model and drug-induced liver injury [29]. PPAR-*α* activation inhibits proinflammatory gene expression in vascular smooth muscle cells (VSMCs) and attenuates development of atherosclerosis [30, 31].

The PPAR-*β*/*δ* gene is located on human chromosome 6p21.1-21.2 [24] and is expressed at relatively high levels in adipose tissue, liver, cardiac and skeletal muscle, brain, kidney, colon, and vasculature [28, 32, 33]. Unlike PPAR-*γ* and PPAR-*α*, PPAR-*β*/*δ* is not easily targeted by currently available drugs because of its ubiquitous expression. Thus, the physiological function of PPAR-*β*/*δ* is far less studied and understood [34]. Nevertheless, PPAR-*β*/*δ* activation is known to increase lipid catabolism in adipose tissue, skeletal muscle, and the heart and has been shown to improve the plasma high-density lipoprotein- (HDL-) cholesterol levels and insulin resistance. Additionally, activation has been shown to induce cell proliferation and differentiation [35] and to limit weight-gain with anti-inflammatory effects in the vessel wall through the inhibition of vascular cell adhesion molecule- (VCAM-) 1 and monocyte chemoattractant protein- (MCP-) 1 expression [36–38].

The PPAR-*γ* gene is located on human chromosome 3p25 [24] and is highly expressed in adipose tissue. PPAR-*γ* plays an essential regulatory role in glucose metabolism, adipocyte differentiation, and lipid storage by controlling the transcription of a number of genes involved in these metabolic processes [6, 15, 39–41]. Some key target genes of PPAR-*γ* include the fat-specific adipocyte protein 2 (aP2; FABP), lipoprotein lipase (LPL), FA translocase (FAT/CD36), FA transport, FA-binding protein, acyl-CoA synthase, glucokinase, glucose transporter type 4 (GLUT4), phosphoenolpyruvate carboxykinase, uncoupling proteins (UCP) 1, 2, and 3, and liver X receptor-*α* (LXR-*α*) [6, 39, 40]. Moreover, PPAR-*γ* also regulates genes involved in insulin signaling and the expression of proinflammatory cytokines, such as tumor necrosis factor- (TNF-) *α* [6, 41]. Most importantly, PPAR-*γ* is a well-recognized cellular target for the antidiabetic thiazolidinediones (TZDs), which sensitize cells to insulin and improve insulin sensitivity and activity [42–44]. However, the associated cardiac hypertrophy in response to PPAR-*γ* may be independent to changes in myocardial insulin signaling [45]. PPAR-*γ* protein stability and transcriptional activity are regulated by covalent modifications, including phosphorylation, ubiquitylation,* O*-GlcNAcylation, and SUMOylation [37, 46]. Importantly, PPAR-*γ* functions as a master switch in controlling adipocyte differentiation and development, and its activation plays an important role in glucose metabolism by enhancing insulin sensitivity [37, 47].

To date, many ligands have been identified that activate and modulate PPAR activity [48]. PPAR ligand-binding activities are 3-4 times greater than that of the other nuclear receptors and thus have the ability to bind a diverse set of synthetic and natural lipophilic acids, such as essential FAs (EFA) [49]. For example, endogenous lipid metabolites from saturated or unsaturated FAs bind nuclear receptors and activate or repress gene expression [48]. Another group of PPAR ligands consists of EFA lipid metabolites—such as arachidonic acid derived from lipoxygenase or cyclooxygenase activity [48]. However, both eicosanoids and EFA are required in relatively high concentrations (~100 *μ*M) for PPAR activation [50]. In particular, the best-characterized endogenous ligands known to stimulate PPAR-*α* are the eicosanoids LT B4 and 8-hydroxyeicosatetraenoic acid (HETE), while 15d-prostaglandin (PG) J2 and 13-hydroxyoctadecadienoic acid (HODE) activate PPAR-*γ* [48]. Other essential FA metabolites, such as 15-HETE, have been suggested to activate PPAR-*β*/*δ* [48]. The physiological roles, expression, gene targets, and ligands of the various PPAR isoforms are summarized in Tables 1 and 2 and the following references [49, 51].

## 4. PPAR Functions in the Cardiovascular System

Many studies have reported on the complex metabolic and biological roles of PPARs in several cardiovascular diseases, including cardiac hypertrophy and heart failure [52–56]. In the cardiovascular system, PPARs have various functions outside of their characteristic roles in metabolism, including extracellular matrix remodeling, oxidative stress, inflammation, and circadian rhythm regulation [57].

Abnormalities in PPAR function have been reported in arrhythmogenic right ventricular dysplasia (ARVD), a rare genetic disease characterized by a progressive fibrofatty infiltration, decreased PPAR-*α*, and increased PPAR-*γ* expression in the right ventricle. The link between PPAR dysfunction and desmosomal genetic mutations is beginning to be understood via Wnt/*β*-catenin pathway analyses [58–61]. PPAR-*γ* is a prime inducer of adipogenesis in ARVD, and the Wnt-*β*-catenin pathway appears to act though a similar mechanism for desmosomal abnormalities [58].

The biological functions of PPAR-*α* in the myocardium have been extensively investigated using PPAR-*α* knockout (KO) mice [62–64]. Despite a normal life span, PPAR-*α* KO mice exhibit progressive cardiac fibrosis with abnormal mitochondria and myofibrils [63]. Histological studies also revealed significant cardiomyocyte hypertrophy [65]. In addition, ex vivo left ventricular papillary muscle exhibits reduced shortening velocity and isometric tension, suggesting that the loss of PPAR-*α* is closely involved in the cardiac dysfunction induced by affecting the impairment of myosin molecule itself, targeting for oxidative stress [65–68]. This is also apparent in echocardiography studies [65]. Interestingly, the development of physiological cardiac hypertrophy, such as is seen after birth and in response to exercise, showed the increased PPAR-*α* expression that parallels an induction of FA utilization [69, 70]. In contrast, PPAR-*α* gene expression is downregulated in the heart of some pathological conditions, especially pressure overload-induced cardiac hypertrophy, that lead to the cardiac lipotoxicity as an accumulation of triglyceride and diacylglycerol [70–73].

The redox system in PPAR-*α* KO mice is subjected to dramatic and/or long-lasting perturbations as well as cardiac dysfunction that appear to result from the direct impairment of myosin II [65]. There is strong evidence that PPAR-*α* activation is necessary to prevent cellular oxidative damage that may occur during physiological cellular metabolism or under conditions of inflammation and oxidative stress, likely caused by repressing NF-*κ*B signaling and limiting inflammatory cytokine production [74, 75]. Therefore, chronic deactivation of the PPAR-*α* signaling pathway may upset the normal equilibrium between oxidant production and antioxidant defenses, which can contribute to cardiac damage [58]. A recent study in PPAR-*γ* KO mice revealed that PPAR-*γ* plays a crucial protective role in cardiomyocytes and may prevent myocardial ischemia-reperfusion injury by modulating NF-*κ*B-associated inflammatory mechanisms in the infarcted myocardium [76].

The heart responds to FA variations by activating PPARs [77]. PPAR-*α* can mediate diurnal variations in the responsiveness of the heart to both FAs and specific PPAR-*α* agonism (WY-14 643) [78]. In the normal heart, however, PPAR-*α* mRNA exhibits only weak circadian oscillations, although the circadian clock within the cardiomyocyte mediates diurnal variations in the responsiveness of the heart to increased workload, according to contractile function and metabolic flux levels [79, 80].

PPAR-*α* overexpression in the mouse myocardium attenuates glucose transporter gene expression and glucose uptake [81]. In myocardium perfused with FA and ketone bodies, the glycolytic rate is decreased and additionally cardiomyocyte-specific PPAR-*α* overexpression leads to an augmentation of triglyceride-derived FAs [82]. PPAR-*α* interferes in pyruvate dehydrogenase kinase (PDK), phosphofructokinase, pyruvate dehydrogenase complex (PDC), and phosphofructokinase (PFK) activities, and the cellular regulation of these proteins is influenced by circadian rhythms [83, 84]. Recently it has been demonstrated that vascular PPAR-*γ* is a peripheral regulator of cardiovascular rhythms that controls circadian variations in blood pressure and heart rate through brain and muscle Arnt-like protein- (BMAL-) 1 [85]. PPAR-*γ* appears to be a main component of the vascular clock. Pioglitazone, a PPAR-*γ* activator, readjusts the circadian rhythm of blood pressure from nondipper to dipper in patients with type 2 diabetes [86]. Accordingly, the impairment of cardiovascular rhythmicity parallels the diurnal variations in urinary excretion of epinephrine and norepinephrine, which are suppressed in PPAR-*γ* mutant mice [85], similar to that observed in BMAL1 KO mice [87].

## 5. PPAR Regulation by Posttranslational Modification in the Myocardium

Energy utilization in heart is transcriptionally controlled in part by the PPAR family and their coreceptors/coactivators, including PPAR-*α*, PPAR-*β*/*δ*, PPAR-*γ*, RXR-*α*, and PPAR-*γ* coactivator- (PGC-) 1*α*. Mechanistically, PPAR-*α*, PPAR-*β*/*δ*, and PPAR-*γ* heterodimerize with the RXR-*α* and coactivators (e.g., PGC-1*α*) and repressors (e.g., nuclear receptor corepressor (NCoR)) to regulate the transcription of genes involved in energy regulation and lipid metabolism [4, 88–90]. Both PPAR and RXR-*α* interact with their respective ligands to enhance PPAR-DNA binding [88, 91]. In the absence of ligand binding, the unbound PPAR-RXR heterodimer remains bound to NCoR and silencing mediator of retinoid and thyroid hormone receptor (SMRT), two main corepressors within the corepressor complex [92, 93]. Both NCoR and SMRT directly interact with the Sin3 complex to form a multisubunit repressor complex [92, 94]. SMRT functions as a protein platform to promote the mobilization of histone deacetylases (HDACs) to the DNA promoters bound by specific interacting transcription factors [92, 94]. Receptor interacting protein- (RIP-) 140, also known as nuclear receptor interacting protein- (NRIP-) 1, is another corepressor that directly recruits HDAC and represses the activity of numerous nuclear receptors including PPARs by competing with their coactivators [95–97]. In the absence of ligand activation of nuclear receptor, the corepressor protein complex is known to suppress target gene transcription by causing the deacetylation of histones [92, 93].

Alterations in the cardiac expression of all three PPARs cause disturbances in glucose and FA metabolism that result in an increased susceptibility to insults or significant dysfunction [91]. While PPAR regulation is known to play a role in cardiovascular disease pathogenesis, the mechanisms regulating their expression and function at the cardiomyocyte level have not been clearly depicted. However, some progress has been made. For example, PPARs may be controlled through posttranslational modifications (PTM), such as SUMOylation and ubiquitination [88]. The conjugation of small ubiquitin-like modifier (SUMO) or ubiquitin is distinctive among PTMs in that it induces the attachment of another polypeptide, rather than the addition of a functional group [88, 98–101]. To date, SUMOylation- or ubiquitination-mediated PPAR regulation in the heart has not been reported; however, PPARs are controlled by these PTMs in other closely related muscle cell types. Other studies have established that SUMOylation of PPAR-*γ*1 promotes VMSC migration and proliferation. This has been demonstrated by using VSMCs transfected with a SUMOylation-defective lysine (K107R) PPAR-*γ*1 mutant, which results in a more potent transcriptional inhibition of inducible nitric oxide synthase when compared to cells transfected with a wild-type construct [88, 102]. These findings regarding the role of PPAR-*γ* SUMOylation in regulating the FA oxidation response and apoptosis in striated muscle and vascular smooth muscle, respectively, provide support for the concept that PPARs could be subjected to posttranslational regulation in the heart. Moreover, PPAR-*α* phosphorylation by the MAPK p38 decreases PPAR-*α* transcriptional activity [88, 103]. Since the p38 pathway is activated in response to cardiac stress—as found in diabetes, heart failure, and cardiac hypertrophy—this study implicates PPAR-*α* activation mechanism by which the heart responds to unfavorable stimuli. The broader implications of these studies indicate that the FA and glucose shifts seen in these diseases may be due to these regulatory mechanisms [88, 104].

## 6. Cardiac Pathophysiology in Genetic Animal Models of PPARs and Their Regulators

### 6.1. PPAR-*α*

The functions of cardiac PPAR-*α* have been evaluated in PPAR-*α* KO mice. While viable and outwardly normal, these mice exhibit mild aging-associated cardiac fibrosis [63]. The basal expression of several PPAR-*α* target genes and rates of FA oxidation are also diminished in hearts of PPAR-*α* KO mice [63, 105, 106] and fail to be induced in response to fasting or diabetes [105]. Moreover, PPAR-*α* KO mice exhibit increased glucose uptake,* GLUT4* expression, and reliance on glucose for cardiac ATP production [64, 107]. Notwithstanding the age-associated fibrosis, cardiac function is relatively normal in young PPAR-*α* KO mice; however, the response to several physiological stressors is perturbed. For example, hearts isolated from PPAR-*α* KO mice are unable to compensate when challenged with an increased workload [64, 108]. Furthermore, transgenic animal models overexpression of PPAR-*α* results in a cardiomyopathy that mimics that seen in diabetes mellitus (DM) [109] that is dependent on dietary fat. This implies that serum-free FA is an essential mediator during cardiac maladaptation [110]. Paradoxically, although chronic exposure to excess FA represses PPAR-*α* expression in cardiomyocytes, this downregulation of PPAR-*α* may result in further myocardial damage by suppressing cellular free FA oxidation on a background of excess free FAs within cells and in the circulation [111]. The PPAR-*α* agonist BM 17.0744 (Roche Pharmaceuticals) normalized cardiac metabolism but was unable to improve cardiac function when given orally to type 2 DM db/db mice for 8 weeks [112]. Apoptosis plays a role in the pathophysiology of diabetic cardiomyopathy and the PPAR-*α* ligand, fenofibrate, was shown to suppress apoptosis. These findings support the potential role of PPAR-*α* ligands in diabetic cardiomyopathy [109, 113].

Cardiovascular PPAR-*α* expression has anti-inflammatory and antioxidative effects, and activation of inflammatory signaling pathways is important in cardiomyocyte hypertrophy [65, 114]. Accordingly, PPAR-*α* agonists have been useful in repressing the inflammation caused by cardiovascular disease. Pretreatment of neonatal cardiomyocytes with PPAR-*α* agonist significantly decreases lipopolysaccharide- (LPS-) stimulated TNF-*α* release, interleukin- (IL-) 1-induced IL-6 secretion, and PG and cyclooxygenase-2 expression [115, 116]. The nuclear translocation of NF-*κ*B and apoptosis were also demonstrated to be reduced after treatment with the PPAR-*α* agonists in the reperfused myocardium. These findings suggest an important role of PPAR-*α* agonists in inhibiting inflammation in many cell types in cardiovascular disease [117, 118]. Moreover, potent PPAR-*α* agonist WY14643 has cardioprotective and cardiodepressive effects when used to treat encephalomyocarditis virus-induced myocarditis in diabetic mice, which may be due to its anti-inflammatory properties and its ability to increase cardiac adiponectin expression, whereas the reduced cardiac efficiency may be due to its enhancement of cardiac UCP3 mRNA expression [6, 119].

### 6.2. PPAR-*β*/*δ*

A decrease in cardiac expression of PPAR-*β*/*δ* was found in rats with diabetic cardiomyopathy [120, 121], and reduction in PPAR-*β*/*δ* expression during hyperglycemia is associated with increased reactive oxygen species production [121], TNF-*α*, IL-6, and nicotinamide-adenine dinucleotide phosphate (NADPH) activity. Further studies are needed to evaluate the precise role of PPAR-*β*/*δ* ligands in regulating diabetic cardiomyocytes [109, 120]. The selective PPAR-*β*/*δ* ligand GW501516 was evaluated for its effect on FA-induced inflammation in cardiomyocytes [122]. GW501516 was also found to reduce expression of the NF-*κ*B target genes, MCP-1 and TNF-*α* in both human cardiac AC16 cells stimulated by palmitate, as well as in the hearts of mice fed with a high-fat diet. This data implies that PPAR-*β*/*δ* may counteract NF-*κ*B activity; thus, PPAR-*β*/*δ* activation might be therapeutically useful as an anti-inflammatory agent in diabetic cardiomyopathies [122].

### 6.3. PPAR-*γ*

In contrast to the induction of the other PPAR family members, there are several studies that revealed that PPAR-*γ* expression is elevated in diabetic rat models [109, 120, 123, 124]. PPAR-*γ* leads to elevations in lipogenic enzymes, which subsequently increase triglyceride production [123]. In addition, recent evidence from animal models showed that cardiomyocyte PPAR-*γ* activation is associated with compromised cardiac function through its lipogenic effects, which may contribute to intracellular triglyceride accumulation and cardiac lipotoxicity [125]. The PPAR-*γ* ligand rosiglitazone may also have a protective role against apoptosis in diabetic cardiomyopathy, similar to the PPAR-*α* ligand [113]. Rosiglitazone has also been demonstrated to decrease cardiac fibrosis and improve left ventricular diastolic dysfunction through the inhibition of receptors for advanced glycated end products and connective tissue growth factor in diabetic myocardium [126]. Moreover, pioglitazone attenuated the deterioration of ischemic preconditioning against reperfusion arrhythmias in type 2 DM rats [127]. Although PPAR-*γ* levels are relatively low in myocardial cells, activation during inflammation might have important effects on cardiomyocytes.

The therapeutic effects of PPAR-*γ* ligands have been attributed primarily to their anti-inflammatory properties. Previous studies showed that both natural and synthetic PPAR-*γ* ligands have anti-inflammatory potentials [128]. The pretreatment of neonatal cardiomyocytes with PPAR-*γ* agonists significantly decreased the LPS-stimulated TNF-*α* release by cardiac myocytes [115]. Moreover, PPAR-*γ* ligands suppressed myocardial mRNA expressions of inflammatory cytokines and IL-1*β* in an autoimmune myocarditis model [129]. Interestingly, treatment with rosiglitazone or pioglitazone decreased the expression of proinflammatory markers and reduced accumulation of neutrophils and macrophages in reperfused myocardium [130, 131]. Nevertheless, high doses of PPAR-*γ* agonists were shown to induce cardiac dysfunction with marked changes in the utilization of free FA and glucose. Thus, the pathophysiological mechanisms on the cardiac effects of PPAR-*γ* agonists causing an increased incidence of myocardial dysfunction are yet to be elucidated [109, 132]. The model of constitutive, whole-body disruption of PPAR-*γ* results in embryonic lethality due to cardiac and placental defects [133], preventing the evaluation of the cardiac phenotype of these mice. However, cardiac-specific PPAR-*γ* (csPPAR-*γ*) KO mice revealed that csPPAR-*γ* deficiency only caused modest ventricular hypertrophy and did not impair systolic function in the unstressed condition [134]. Increased PPAR-*γ* expression was found in the spontaneously hypertensive rat that may have resulted from increased lipid uptake or as a compensatory response to cardiac hypertrophy and failure, thereby compromising cardiac function [124, 125].

## 7. Therapeutic Outcomes of PPAR Ligands in Heart Disease

### 7.1. PPAR-*α* Agonists

Synthetic PPAR-*α* ligands—such as clofibrate, fenofibrate, and bezafibrate—decrease triglyceride-rich lipoproteins through an increase in the gene expression of FA-*β*-oxidation and decrease in the expression of apolipoprotein (Apo) C-III [135, 136]. The above-noted drugs are extensively used in the treatment of hypertriglyceridemia. Such fibrates not only have a triglyceride-lowering effect, but also increase HDL-cholesterol levels resulting from the increase in the expressions of ApoA-I and ApoA-II [135–137].

Human trials with PPAR-*α* agonists have largely, but not uniformly, supported possible atherosclerotic benefits. In the Bezafibrate Coronary Atherosclerosis Intervention Trial (BECAIT), bezafibrate treatment decreased angiographic evidence of coronary atherosclerosis [138, 139]. In the Helsinki Heart Study (HHT), gemfibrozil decreased cardiovascular events, especially among patients with diabetes, but an increased rate of noncoronary death was also noted [140]. In the Bezafibrate Infarction Prevention (BIP) trial, only the subgroup with the highest triglyceride levels showed a decrease in adverse cardiovascular events with fibrate therapy [141]. In the Veteran's Administration-HDL Intervention Trial (VA-HIT), gemfibrozil treatment showed a statistically significant decrease in cardiovascular events in the cohort with average LDL-cholesterol levels, history of cardiovascular disease, and modestly decreased HDL-cholesterol/elevated triglycerides [142–144]. Of note, VA-HIT subjects were not on any 3-hydroxy-3-methylglutaryl coenzyme A reductase inhibitors (statins); therefore, the outcomes of this trial may have been driven largely by the effect of gemfibrozil in patients with insulin resistance and/or diabetes [143, 144]. The Fenofibrate Intervention and Event Lowering in Diabetes (FIELD) study—a large, randomized, placebo controlled trial—investigated the effects of fenofibrate on first or recurrent cardiovascular events in patients with type 2 diabetes and found that the primary end point did not achieve a statistically significant difference between treatment groups. Several secondary end points were significantly reduced, including total cardiovascular events and nonfatal myocardial infarction. Somewhat surprising was the finding that decreases in small-vessel diseases; namely, nephropathy and retinopathy were also found. An increase in cardiovascular mortality also was noted with fenofibrate but did not reach statistical significance [145]. Comparing the positive outcomes of VA-HIT with gemfibrozil, a less potent PPAR-*α* agonist, to the negative results seen in FIELD achieved with fenofibrate, a more potent PPAR-*α* agonist might support PPAR modulation, as opposed to more powerful activation, as being clinically effective. More potent PPAR binding may not necessarily correlate with greater clinical advantage, particularly because PPAR agonists have been defined mainly in vitro [138, 146, 147].

Importantly, FIELD does not establish the impact of statin plus fibrate combination therapy on cardiovascular disease. Thus, the hypothesis that combination of a statin plus a fibrate might offer greater cardiovascular risk reduction than a statin alone implies the requirement of another clinical study such as the Action to Control Cardiometabolic Risk in Diabetes (ACCORD) trial. However, ACCORD-lipid arm in patients with DM did not demonstrate any reduction in fatal cardiovascular incidences or nonfatal myocardial infarction and stroke compared with simvastatin alone [148]. From the disappointing cardiovascular outcomes in these studies, we might expect VA-HIT and FIELD to specify advantages of fibrates in patients who are statin intolerant or for possible fibrate benefits to microvessel disease, which is a major source of morbidity in diabetes [138]. Furthermore, prespecified subgroup analysis of the ACCORD data suggested a possible benefit of fenofibrate in patients with high triglyceride and low HDL-cholesterol baseline levels. Therefore, fibrates may prove to be beneficial in treating atherogenic dyslipidemia in diabetes patients [51, 148].

### 7.2. PPAR-*γ* Agonists

PPAR-*γ* is a regulator of glucose and lipid metabolism; therefore, its synthetic PPAR-*γ* ligands—such as glitazones and TZD derivatives (such as troglitazone, rosiglitazone, and pioglitazone)—improve glucose and insulin parameters and increase whole body insulin sensitivity. Therefore, they are called insulin-sensitizers and are used in the treatment of diabetes [149]. In early human trials, PPAR-*γ* agonists showed decreased in-stent restenosis after coronary stent implantation [150, 151]. Furthermore, in the Carotid Intima-Media Thickness in Atherosclerosis Using Pioglitazone (CHICAGO) study, significant effects of pioglitazone on the slow progression of carotid intima-media thickness were reported in patients nearly matched for glycemic control with glimepiride [152].

Rosiglitazone and pioglitazone are used in the treatment of patients with type 2 diabetes; however, the effects of these TZDs on cardiovascular outcomes in patients with DM are different. The Prospective Pioglitazone Clinical Trial in Macrovascular Events (PROactive) trial investigated the effects of pioglitazone combined with standard contemporary antidiabetic treatment versus active, but non-TZD, antidiabetic treatment on a combined vascular end point in individuals with known macrovascular disease [153]. The purpose of the PROactive study was to achieve similar, matched hemoglobin A1c (HbA1c) levels in the TZD and non-TZD groups in order to provide more definitive insights into glucose-independent vascular effects of TZDs. In spite of the extensive in vivo and in vitro data supporting TZD effects on atherosclerosis, no statistically significant difference was noted in the primary end point between study groups. In contrast, the main secondary end point was revealed with a statistically significant 16% decrease in clinical events [153, 154]. Contrary to pioglitazone, rosiglitazone was associated with significant increases in death from cardiovascular causes and myocardial infarction after a relatively short-term of exposure [155]. Thus, the European Medicines Agency withdrew approval of rosiglitazone in 2010 due to these cardiovascular safety concerns [156]. Importantly, these divergent outcomes may result from their diverse effects on lipid subfractions [157]. Pioglitazone increases HDL-cholesterol and decreases fasting plasma free FAs and triglycerides without any influence on total cholesterol and LDL-cholesterol; however, rosiglitazone significantly augments HDL-cholesterol levels, as well as total cholesterol and the LDL-cholesterol fraction levels [156, 158, 159].

In the Diabetes Reduction Approaches with Ramipril and Rosiglitazone Medications (DREAM) study, the effects of the angiotensin-converting enzyme inhibitor ramipril and rosiglitazone on the prevention of diabetes were studied using a two-by-two placebo-controlled design [160]. Interestingly, rosiglitazone significantly reduced the progression to diabetes in a cohort with impaired fasting glucose and/or impaired glucose tolerance, whereas ramipril had no effect on this measure [161]. The Actos Now for the Prevention of Diabetes (ACT NOW) trial analyzed with a similar question to DREAM in patients with impaired glucose tolerance randomized to receive either pioglitazone (45 mg) or placebo. After a mean follow-up of 2.2 years, progression to diabetes occurred in 5% of the pioglitazone group, compared with 16.7% of the placebo group, but too few cardiovascular events occurred (pioglitazone 26, placebo 23) to draw any inferences regarding effect of treatment on cardiovascular outcomes [162, 163]. This decrease in diabetes progression with pioglitazone was consistent with previous studies, including the troglitazone arm of the Diabetes Prevention Program and women with a history of gestational diabetes [164, 165]. Additionally, the pioglitazone arm of the Pioneer study revealed significantly greater improvements in inflammatory markers—including high-sensitivity CRP, MMP-9, and MCP-1—than the glimepiride-treated group despite equivalent reductions in fasting glucose and HbA1c levels. In an additional subgroup analysis, patients with no significant glucose responses to pioglitazone still had improved surrogate markers for atherosclerosis. Despite limitation by the small numbers of patients in these subgroups, such findings continue to raise possible disassociations between TZD-mediated effects on the vasculature and inflammation versus its glycemic advantages [138, 166].

In the Cardiovascular Outcomes in Oral Agent Combination Therapy for Type 2 Diabetes (RECORD) trial, 4,447 subjects with type 2 DM poorly controlled on monotherapy with metformin or sulfonylurea, a noninferiority hypothesis was explored for rosiglitazone as second-line therapy in type 2 diabetes [163, 167]. The primary end point of RECORD was time to cardiovascular hospitalization or cardiovascular death. After a mean follow-up of 5.5 years, primary endpoint events occurred in 321 patients in the rosiglitazone group and 323 patients in the metformin/sulfonylurea group, thus meeting the requirement for noninferiority of rosiglitazone. Fatal or nonfatal HF occurred more frequently in the rosiglitazone group than in the active control group (61 versus 29 patients). Limitations of RECORD include an event rate that was substantially lower than that projected in trial design with consequent reduction of statistical power, and potential complications resulting from the differential use of statins and diuretics, and an open-labeled study design [163, 167].

Despite many beneficial features of glitazones, they also exhibit adverse effects, such as edema, heart failure, weight gain, bone fractures, and increased risk of myocardial infarctions, which have limited the use of TZDs in diabetic patients with high lipid levels [168]. In the PROactive study, an increased incidence of congestive HF was reported in the pioglitazone group, although these events were not well judged. Previous work has clearly demonstrated that TZDs can cause fluid retention, as evident from the modest decrease in hematocrit and volume expansion documented with TZD exposure [169]. The incidence of pedal edema observed with TZD monotherapy is about 3% to 5% compared with 1.2% in placebo arms [170]. The incidence of pedal edema with TZDs approaches 7.5% when combined with either metformin or sulfonylurea, compared with 2.5% and 2.1% with sulfonylurea or metformin alone, respectively [171]. The risk of pedal edema appears similar with both rosiglitazone and pioglitazone in clinical use [172]. Concomitant insulin and TZD use has been associated with a 2- to 3-fold higher rate of edema compared to insulin alone, with rates increasing from 5% to 7% with insulin alone to 13% to 15% with TZD and insulin [171]. Recent data suggest that upregulation of a specific sodium channel—sodium channel, nonvoltage gated 1 gamma subunit (SCNN1G)—in the distal nephron is a PPAR-*γ*-mediated mechanism for TZD-induced edema [173, 174]. Other mechanisms involved for TZD-mediated edema include altered interstitial ion transport, increased sympathetic nervous system activity, and altered endothelial permeability [175–177]. This edema is reversible and should not necessarily be equated with myocardial toxicity although some patients with DM, even absent class III or IV HF, may not tolerate this volume expansion [138].

Another clinically significant side effect of TZDs is body weight gain. This change, which likely involves both fluid retention and increases in adiposity, is typically in the range of 2 to 5 kg [178]. Some of the weight induced by TZDs may be advantageous, involving a shift from visceral to subcutaneous areas, and also track the increase in adiponectin, anti-inflammatory protein, induced by TZDs [179]. The change in fat distribution with TZDs includes a change in energy balance and possible effects on other factors and pathways influencing body weight, because a simple rearrangement in fat location would not explain an overall net increase in body mass [138, 180]. Nevertheless, the weight increase seen with PPAR-*γ* activation has clearly contributed to the hesitation of TZDs usage as antidiabetic drug, which may be more serious when combined with insulin [181].

### 7.3. PPAR-*α*/*γ* Dual Agonists

A new class of dual PPAR-*α*/*γ* agonists has been shown to have a positive influence on both glucose and lipid metabolism and are currently under development as a response to the treatment challenge of coexisting type 2 diabetes with dyslipidemia. These dual agonists not only reduce arteriosclerosis development, but also have an antidiabetic capacity. They also exhibit improvement of endothelial function, anti-inflammatory, and anticoagulant action, decrease plasma free FAs, and lower blood pressure, indicative of advantageous effects on the vasculature [49].

Until now, several attempts to develop a dual agonist for diabetes have failed due to various safety concerns: ragaglitazar, MK-0767, and naveglitazar were all found to be associated with an increased incidence of bladder cancer and hyperplasia in rodent studies [51, 182], and tesaglitazar development was discontinued due to indications that it may cause renal dysfunction [183]. The most-studied dual agonist muraglitazar was found to be effective in reducing HbA1c and triglyceride levels while increasing HDL-cholesterol levels [51, 184–188]. One randomized, double-blind trial of 1,477 drug-naive patients with type 2 diabetes found a −0.25% to −1.76% (3–17 mmol/mol) reduction in HbA1c from baseline after 24 weeks of muraglitazar treatment, compared with a reduction of −0.57% (5 mmol/mol) with pioglitazone [51, 186]. At 12 weeks, triglycerides had decreased by −4 to −41% with muraglitazar and 9% with pioglitazone and HDL-cholesterol had increased by 6–23% with muraglitazar and 10% with pioglitazone. Nevertheless, Bristol-Myers Squibb discontinued further development of this dual agonist in 2006 after Nissen and colleagues published an analysis of the available material from the clinical trial program, which revealed that muraglitazar was associated with an increased incidence of the composite end point of death, major adverse cardiovascular events, congestive HF (relative risk: 2.62; *P* = 0.04), and excessive morbidity for all individual components of the composite endpoint when compared to placebo or pioglitazone [51, 188].

Aleglitazar (Hoffmann-La Roche) is the most recent dual PPAR-*α*/*γ* agonist that has completed in phase III trials and has a balanced affinity for both PPAR-*α* and PPAR-*γ* receptor subtypes. Preclinical and clinical trial results have been promising [51, 189–192]. Phase II study SYNCHRONY has shown a significant dose-dependent reduction in HbA1c of −0.36% (4 mmol/mol, 50 *μ*g; *P* = 0.048) to −1.35% (15 mmol/mol, 600 *μ*g; *P* < 0.0001) after 16 weeks of treatment with aleglitazar once daily when compared with placebo. Importantly, statistically significant beneficial effects on lipid subfractions were also found. Significant decreases in triglyceride (*P* < 0.001 for percentage changes) and increases in HDL-cholesterol (*P* < 0.05 for percentage changes) were found with all doses of aleglitazar (−43 and +21%, resp., with the 150 *μ*g dose). In addition, significant reductions in LDL-cholesterol were found at doses of 150 *μ*g or higher, compared with placebo (*P* < 0.05 for percentage changes): placebo-adjusted reduction in LDL-cholesterol with the 150-*μ*g dose of aleglitazar was −15.5%. Indeed, aleglitazar, at the 150-*μ*g dose, was associated with a greater effect on triglycerides, HDL-cholesterol, and LDL-cholesterol than pioglitazone 45 mg. Further analysis of this study data suggests that aleglitazar produces a shift from the atherogenic small dense LDL particles associated with type 2 diabetes to larger LDL particles [51, 193]. Phase III study ALECARDIO, randomized double-blind placebo-controlled clinical trial, had evaluated the hypothesis that aleglitazar (150 *μ*g daily dose) can reduce cardiovascular mortality and morbidity in patients with type 2 DM who have suffered from a recent acute coronary syndrome (ACS) event. However, use of aleglitazar in patients with type 2 diabetes and recent ACS did not significantly reduce the incidence of cardiovascular death, myocardial infarction, or stroke. Unfortunately, aleglitazar increased the risks of HF, renal dysfunction, bone fractures, gastrointestinal hemorrhage, and hypoglycemia [194].

There are several potential explanations for why aleglitazar did not reduce cardiovascular mortality and morbidity in ALECARDIO trial. First, the magnitude of changes in HDL-cholesterol and triglyceride levels achieved with aleglitazar may not be sufficient to impart additional cardiovascular benefits when administered concurrently with statins. Second, some therapies may be unable to exert a cardioprotective effect in patients with extensive atherosclerosis and long-standing diabetes or may require a very long duration of exposure to achieve such effects. Third, favorable lipid and metabolic effects of aleglitazar may have been negated by adverse effects of the drug, including heart failure, reduced renal function, hypoglycemia, and increased LDL-cholesterol, resulting in no net cardiovascular benefit. These findings do not support the use of aleglitazar in this setting with a goal of reducing cardiovascular risk [51, 194].

## 8. New Modalities and Future Directions of PPAR-Directed Therapeutics

The impact of fibrates and TZDs on dyslipidemia and diabetes is linked primarily to PPAR-*α* and PPAR-*γ* activation, respectively [195, 196]. However, substantial clinical and preclinical experience has shown that individual drugs differ from one another in therapeutic and side effect properties [42, 197]. Furthermore, PPAR expression in multiple tissues raises the possible value of targeting PPAR agents in therapeutic indications of a number of other diseases (e.g., cancer and colitis) [122, 198–201]. Although many clinical studies of PPARs have demonstrated inconsistent results for cardioprotective effects [139–141, 145, 153, 167, 193, 194], the evidence reviewed above suggests that this is still a lucrative area of study. Therefore, the needs of new PPAR-directed therapeutic modalities must include pan-PPAR agonists, selective PPAR modulators, dual PPAR agonists, PPAR-*γ* antagonists, and nutraceuticals, all of which are being considered as possible approaches to reduce the adverse events seen with current TZDs [138, 181, 202].

### 8.1. Pan-PRAR Agonists

The significant structural similarity of PPAR-*α*, PPAR-*β*/*δ*, and PPAR-*γ*—particularly within their ligand-binding domains—has allowed the identification of several synthetic dual- or pan-PPAR agonists [203]. Active metabolites of fibrates, such as fenofibric acid and clofibric acid, are dual activators of PPAR-*α* and PPAR-*γ*, with about a 10-fold selectivity for PPAR-*α*. Another compound from this group, bezafibrate, is a broader activator because it activates all three PPAR subtypes at comparable doses to other fibrates. Therefore, bezafibrate is regarded as a pan-agonist with the potential to directly improve insulin sensitization via PPAR-*γ* activation [10, 15].

### 8.2. Selective Modulators and Partial Agonists

The intensive search for safer PPAR agonists led to the development of selective partial PPAR modulators. Currently, new selective PPAR-*γ* modulators are in development—including S26948 [204] and INT131 [205], which should stimulate glucose metabolism and minimize the adverse effects of full PPAR-*γ* agonists [49]. INT131 recruits vitamin D3 receptor interacting protein- (DRIP-) 205 and promotes its binding to a level of approximately 30% of that conferred by the full PPAR-*γ* agonist rosiglitazone [206]. In animal models of diabetes, INT131 caused less weight gain compared to pioglitazone or rosiglitazone while retaining efficacy to reduce plasma glucose [206, 207]. Importantly, toxicity of INT131 in cynomolgus monkeys and rats was not associated with fluid retention, changes in hematocrit, or weight gain over 6 months [207, 208]. In a phase II study, however, INT131 was associated with an increase in the incidence of edema, weight gain, and decreased hematocrit at the 10 mg dose versus placebo, highlighting the difficulty in translating promising preclinical profiles into patients [209]. While the cardiac adverse effect profile of rosiglitazone-like PPAR-*γ* full agonists is unfortunate, the therapeutic potential of novel pharmacological agents targeting PPAR-*γ* submaximal cannot be excluded. Interestingly, newly synthesized partial PPAR-*γ* agonists, such as balaglitazone, MBX-102, MK-0533, PAR-1622, PAM-1616, KR-62776, and SPPAR-*γ*M5, have a reduced tendency to cause the adverse effects associated with full PPAR-*γ* agonists or may be entirely devoid of such effects [6, 47].

### 8.3. Phosphorylation and Posttranslational Control

As noted above several compelling new mechanisms of posttranslational control of PPAR action have recently been described, including phosphorylation, SUMOylation, ubiquitination, and nitration [210]. In addition to enhancing the transcriptional activity of PPAR-*γ*, rosiglitazone was found to inhibit the PPAR-*γ* phosphorylation at Ser273 by cyclin-dependent kinase 5 (CDK5) in adipose tissue, preserving the transcription of insulin-response genes and correlating with antidiabetic activity. A second PPAR-*γ* agent, MRL24, was as effective as rosiglitazone at blocking phosphorylation and improving diabetes in animal models, despite being only a partial PPAR-*γ* agonist. Taken together, these results suggest that the insulin-sensitizing benefits of PPAR-*γ* agonists are due in part to their ability to block phosphorylation and not solely to their agonist activity [211].

### 8.4. Nongenomic Regulation

Recent evidence also suggests the potential role of nongenomic regulation of PPAR-*γ* and PPAR-*α*, mediated by interaction with cytosolic second messengers, including kinases and phosphatases [210]. The MAP/ERK kinase, MAPK kinase- (MEK-) 1, was reported to bind directly to the AF-2 domain of PPAR-*γ* in response to mitogenic stimulation, leading to the sequestration of PPAR-*γ* in the cytoplasm [212]. Selective inhibition of MEK-1/PPAR-*γ* interactions has recently been proposed as a concept for treatment of cancer, inflammation, and metabolic disorders but has yet to gain significant acceptance [212].

### 8.5. New Dual PPAR-*α*/*γ* Agonists

Saroglitazar, a PPAR agonist with predominant PPAR-*α* and moderate PPAR-*γ* activity, was launched exclusively in India for the control of dyslipidemia [213, 214]. However, limited data is available on its molecular profile, and the treatment duration and low patient number in its phase III program make it impossible to draw conclusions regarding its cardiovascular and long-term safety profiles [203].

### 8.6. Nutraceuticals and Life-Style Modification

As endogenous nuclear receptor ligands, dietary n-3 and n-6 polyunsaturated FAs (PUFAs) and their derivatives can upregulate PPAR-*γ* expression in vitro and in vivo and reduce an inflammatory response [215]. Furthermore, it has been shown that any type of regular exercise and crataegus species would improve cardiovascular function and minimizes several risk factors via stimulating lipid metabolism by acting on enzymes and genes expression such as ATP-binding cassette transporter A1 (ABCA1) and PPAR-*α* which are involved in this process [216]. However, though dietary PUFAs similar to synthetic ligands were able to bind to the ligand-binding domain and cause conformational changes to activate the receptor, they are considered as weak PPAR-*γ* ligands because of their low physiological concentrations. Another caution of nutraceuticals is that some of the flavonoids have been associated with tumor and altering pharmacodynamics and pharmacokinetics of various drugs via interacting with cytochrome P450 enzymes [202].

## 9. Conclusions

PPARs are critical gene regulators in cardiomyocytes, yet their functions are not fully established. PPAR agonists convey beneficial effects as therapeutic agents for diabetes and atherosclerosis by lowering blood glucose, improving insulin resistance, inflammation, and lipid metabolism; however, adverse side effects limit their clinical use. As such, the future of PPAR-directed agents in cardiometabolic therapy remains uncertain, although several late-stage molecules may still hold promise [203]. Future directions in PPAR agonist development are likely to focus on optimizing the PPAR subtype interaction profile, maximizing the inhibition of PPAR-*γ* phosphorylation, and screening against off-target activity. At the present time, clinicians should keep in mind the risk/benefit ratio of PPAR activators. Intensive research on this therapeutic target will likely lead to the development of safer and more effective PPAR agonists in the near future.

## Figures and Tables

**Table 1:** The expression of the PPARs and their gene targets. Modified from [49, 51].

Properties	PPAR-*α*	PPAR-*β*/*δ*	PPAR-*γ*
Tissue expression	*Main tissues*: tissues exhibiting high catabolic rates of FA (liver, skeletal muscle) *Other tissues*: heart, intestine, kidney, and brown adipose tissue	Ubiquitous: however, the biggest expression is in liver, esophagus, intestine, kidney, and skeletal muscle	*Main tissue*: adipose tissue (white and brown) *Other tissues*: liver, intestine, kidney, retina, immunologic system (bone marrow, lymphocytes, monocytes, and macrophages), and trace amounts in muscles

Gene targets	*β*-oxidation pathway (acyl-CoA oxidation, bifunctional enzyme, and thiolase) Sterol 12-hydroxylase (CYP8B1) FATP FAT/CD36 L-FABP Lipoprotein lipase apo A-I and A-II	Genes involved in lipid uptake; it represses genes implicated in lipid metabolism and efflux	FA-binding protein (aP2) Phosphoenolpyruvate carboxykinase (enzyme of the glyceroneogenesis pathway) FATP FAT/CD36

FA: fatty acid, FATP: fatty acid transport protein, L-FABP: liver cytosolic fatty acid-binding protein, and apo: apolipoprotein.

**Table 2:** The natural and synthetic ligands of the PPARs and their physiological roles. Modified from [49, 51].

Properties	PPAR-*α*	PPAR-*β*/*δ*	PPAR-*γ*
Natural ligands	Unsaturated FA, PG, and LT B4 8-Hydroxyeicosatetraenoic acid	Unsaturated FA Carbaprostacyclin Components of VLDL	Unsaturated FA 15-Hydroxyeicosatetraenoic acid 9- and 13-hydroxyoctadecadienoic acid 15-Hydroxy delta 12,14-PG J2 PG J2

Synthetic ligands	Clofibrate and fenofibrate Gemfibrozil	GW501516	Rosiglitazone and pioglitazone Troglitazone and ciglitazone Farglitazar, S26948, and INT131

Physiological roles	Lipid catabolism and homeostasis (stimulating *β*-oxidation of fatty acids), increased breakdown of TG and FA, increased cellular FA uptake, reduced TG and FA synyheis, control of inflammatory processes, and vascular integrity mediate the hypolipidemic function of fibrates *Liver*: increasing FA oxidation and uptake and increasing apoA-I, apoA-II, and HDL *Vessel*: increasing TG, HDL, ABCA1, and apoE and decreasing FFA, VLDL, cytokines, and NF-*κ*B	Dyslipidemia? Wound healing? Increasing fat oxidation in skeletal and cardiac muscle responsible for insulin sensitivity and glucose homeostasis and vascular integrity *Adipocentric action*: decreasing cytokines, resistin, fFFA, and NF-*κ*B and increasing ABCA1 and GLUT4 *Skeletal muscle*: increasing glucose uptake and glycogen synthesis	*Glucose homeostasis and lipid storage*: differentiation and maturation of adipocytes Increasing IS and glucose homeostasis (it prevents hyperglycemia) and vascular integrity *Skeletal muscle/liver/adipocyte*: increasing FA oxidation, UCP, and HDL and decreasing TG

FA: fatty acid; apo: apolipoprotein, PG: prostaglandin, LT: leukotriene, TG: triglyceride, HDL: high-density lipoprotein, ABCA1: ATP-binding cassette subfamily A member 1, FFA: free fatty acid, VLDL: very low-density lipoprotein, NF-*κ*B: nuclear factor kappa-light-chain-enhancer of activated B cells, GLUT4: glucose transporter type 4, and UCP: uncoupling protein.

## Abbreviations

ACS: Acute coronary syndrome
AF: Activation function
Apo: Apolipoprotein
BMAL-1: Brain and muscle Arnt-like protein-1
CDK5: Cyclin-dependent kinase 5
csPPAR-*γ*: Cardiac-specific PPAR-*γ*
DM: Diabetes mellitus
DRIP: Vitamin D3 receptor interacting protein
EFA: Essential FAs
FA: Fatty acid
GLUT4: Glucose transporter type 4
HDAC: Histone deacetylase
HDL: High-density lipoprotein
HETE: Hydroxyeicosatetraenoic acid
HF: Heart failure
HODE: Hydroxyoctadecadienoic acid
IL: Interleukin
KO: Knockout
LPS: Lipopolysaccharide
LT: Leukotriene
LXR-*α*: Liver X receptor-*α*
MCP: Monocyte chemoattractant protein
MEK: MAPK kinase
mRNA: Messenger RNA
NCoR: Nuclear receptor corepressor
NRIP: Nuclear receptor interacting protein
PG: Prostaglandin
PGC: PPAR-*γ* coactivator
PPAR: Peroxisome proliferator-activated receptor
PPRE: Peroxisome proliferator response element
PTM: Posttranslational modification
PUFA: Polyunsaturated fatty acid
RIP: Receptor interacting protein
RXR: Retinoid X receptor
SMRT: Silencing mediator of retinoid and thyroid hormone receptor
SUMO: Small ubiquitin-like modifier
TNF: Tumor necrosis factor
TZD: Thiazolidinedione
VLDL: Very low-density lipoprotein
VSMC: Vascular smooth muscle cell.
